# Intelligence, Correspondence, &c.

**Published:** 1829-10-01

**Authors:** 


### INTELLIGENCE, CORRESPONDENCE, &c.

### DEATH OF MRS. DENMARK.

MRS. DENMARK, respecting whose case there have been so many investigations and conjectures, has died at length. The surgeon, Mr. H———, under whose care she had been originally, and who, it is very well known, has attended her lately, was anxious of course to be present at the post-mortem examination. We learn, however, from a paragraph on the subject in the LONDON MEDICAL GAZETTE, that it was only after some unexpected objections, from what quarter is not stated, that he obtained permission to attend. "The reader," says our contemporary, "may judge of his astonishment, when, on arriving, he found that the dissection had already been completed by Mr. Wardrop!—owing, it was stated, to this gentleman not having received his message."

